# Flublok Quadrivalent Vaccine Adjuvanted with R-DOTAP Elicits a Robust and Multifunctional CD4 T Cell Response That Is of Greater Magnitude and Functional Diversity Than Conventional Adjuvant Systems

**DOI:** 10.3390/vaccines12030281

**Published:** 2024-03-07

**Authors:** Chantelle L. White, Maryah A. Glover, Siva K. Gandhapudi, Katherine A. Richards, Andrea J. Sant

**Affiliations:** 1 David H. Smith Center for Vaccine Biology and Immunology, Department of Microbiology and Immunology, University of Rochester, Rochester, NY 14642, USA; chantelle_white@urmc.rochester.edu (C.L.W.); glove208@umn.edu (M.A.G.); katherine_skelly@urmc.rochester.edu (K.A.R.); 2 Department of Microbiology, Immunology and Molecular Genetics, University of Kentucky School of Medicine, Lexington, KY 40508, USA; siva.gandhapudi@uky.edu

**Keywords:** adjuvants, CD4+ T cells, vaccination, influenza, hemagglutinin

## Abstract

It is clear that new approaches are needed to promote broadly protective immunity to viral pathogens, particularly those that are prone to mutation and escape from antibody-mediated immunity. CD4+ T cells, known to target many viral proteins and highly conserved peptide epitopes, can contribute greatly to protective immunity through multiple mechanisms. Despite this potential, CD4+ T cells are often poorly recruited by current vaccine strategies. Here, we have analyzed a promising new adjuvant (R-DOTAP), as well as conventional adjuvant systems AddaVax with or without an added TLR9 agonist CpG, to promote CD4+ T cell responses to the licensed vaccine Flublok containing H1, H3, and HA-B proteins. Our studies, using a preclinical mouse model of vaccination, revealed that the addition of R-DOTAP to Flublok dramatically enhances the magnitude and functionality of CD4+ T cells specific for HA-derived CD4+ T cell epitopes, far outperforming conventional adjuvant systems based on cytokine EliSpot assays and multiparameter flow cytometry. The elicited CD4+ T cells specific for HA-derived epitopes produce IL-2, IFN-γ, IL-4/5, and granzyme B and have multifunctional potential. Hence, R-DOTAP, which has been verified safe by human studies, can offer exciting opportunities as an immune stimulant for next-generation prophylactic recombinant protein-based vaccines.

## 1. Introduction

There is a critical need to develop more effective vaccine strategies to induce broadly protective immunity to the influenza virus [1,2,3,4] and other highly mutable pathogens such as SARS-CoV-2 [5,6,7,8]. The nature of B cell antibody responses, the target of most vaccine strategies, makes effective and long-term protective immunity to these mutable pathogens extremely challenging. First, the B cell response is largely restricted to epitopes displayed on the three-dimensional structure of the intact protein, and second, among those B cell epitopes, there is well-documented immunodominance to selected sites [5,9,10,11]. In the face of immune pressure from vaccine-elicited antibodies, these sites mutate and escape from the protective effects of the antibodies. Such immune escape is prevalent and well documented in both the influenza virus [12,13,14] and SARS-CoV-2 [15,16,17].

For influenza, multiple approaches have been used to redirect the B cell response toward the genetically conserved regions of HA [18,19,20,21]. These strategies include vaccine constructs comprising the stalk domain ([22] and reviewed in [23]) or the use of chimeric HA constructs with rare HA head domains assembled with the conserved stalk, aimed at focusing the B cell repertoire on conserved epitopes expressed by seasonal and potentially avian influenza HA proteins [24,25]. There are also molecular strategies that seek to induce HA head-specific antibodies that are expected to protect against a variety of influenza viruses expressing diverse HA antigens [26,27,28,29]. Finally, to enhance antibody responses, adjuvants have been explored for their potential to promote the breadth and efficacy of antibody responses to both licensed and novel vaccines [30,31].

Rather than an exclusive focus on vaccine strategies that elicit protective antibody responses, a complementary approach that we and others advocate is to develop and test vaccination approaches that elicit pathogen-specific, protective CD4+ T cells [32,33,34,35,36,37]. Beyond their effector functions, the broad epitope specificity of CD4+ T cells includes peptides from both conserved internal proteins and surface-expressed, more variable proteins (reviewed in [38,39,40,41]). Combined, these features of recognition ensure relatively persistent reactivity and effector function in the face of antibody-mediated mutation evasion strategies. Along with the diverse epitope specificity of CD4+ T cells, it is now well accepted that CD4+ T cells can convey a multitude of discreet functions important to protective immunity. Among these functions is the well-known ability of CD4+ T cells to potentiate the production of Immunoglobulin (Ig)-class-switched high-affinity antibody responses by B cells (reviewed in [42,43,44]). Beyond helping B cells, CD4+ T cells promote CD8 T cell expansion and memory through the production of IL-2 [45] and produce cytokines with direct anti-viral function, such as IFN-γ [46]. Finally, CD4+ T cells can possess cytotoxic activity [47,48,49] that can recognize infected or antigen-bearing antigen-presenting cells (APCs) [49,50,51]. Because of this increasingly recognized role of cellular components of the immune system in providing host protection, there is now a particular interest in enhancing cellular immunity to vaccination with the addition of adjuvants for both cancer immunotherapy and infectious diseases [52,53,54,55].

In this study, we comprehensively examined the impact of adjuvants in promoting recruitment of HA-specific CD4+ T cells during the primary response to a recombinant protein-based licensed influenza vaccine. Although some previous studies have demonstrated the ability of adjuvants to enhance elicitation of cytokine-producing CD4+ T cells, these have typically involved measurements of single mediators and prime boost vaccination regimens. Here, we sought to identify and quantify the multifunctionality of elicited CD4+ T cells after a single vaccination, as evidenced by the simultaneous production of different cytokines and cytotoxic potential by the elicited CD4+ T cells. We were particularly excited to explore the ability of a newly developed enantiospecific cationic lipid nanoparticle (R-DOTAP), promising as an antigen delivery system that has demonstrated safety in Phase I and Phase II clinical trials (NCT02065973, NCT04260126, NCT04580771, NCT05232851) [56]. In animal models, R-DOTAP has primarily been studied for CD8 T cell responses and tumor-specific immunity [57,58,59,60] and, more recently, for elicitation of other T cells and antibodies [61,62]. We hypothesized that the properties of R-DOTAP would also enhance CD4+ T cell responses and differentiation to recombinant protein antigens. These include enhanced uptake of protein antigens into APCs, dendritic cell activation, and the induction of chemokine expression [58,63]. Published studies indicate that R-DOTAP induces Type I IFN responses via MyD88 and endosomal TLR-7 and TLR-9 [59]. Moreover, broadly reactive IgG antibody responses to SARS-CoV-2 and HA recombinant proteins were elicited in response to R-DOTAP vaccination in prime boost strategies [62]. These arrays of features suggest that R-DOTAP is a promising candidate for inducing CD4+ T cell responses that could convey protective immunity to pathogens such as influenza. We recently studied the ability of R-DOTAP and other adjuvant systems in combination with a recombinant HA-B antigen to elicit the cytokines IL-2 and IFN-γ from elicited CD4+ T cells and found that the most robust response was conveyed by R-DOTAP [61].

In the current study, we evaluated the ability of R-DOTAP to promote the elicitation of epitope-specific CD4+ T cells to a licensed recombinant protein-based influenza vaccine (Flublok) after a single vaccination. Traditional squalene-based adjuvants as well as no added adjuvants were used as comparators. Through a preclinical mouse model of subcutaneous vaccination in B6 mice, we discovered striking potentiation in the elicitation of antigen-specific CD4+ T cells in the primary response to R-DOTAP, relative to control adjuvants or the unadjuvanted Flublok vaccine. The elicited CD4+ T cells were multifunctional and produced the cytokines IL-4/5, IFN-γ, and IL-2, as well as the cytotoxic mediator granzyme B. Moreover, the use of multiparameter flow cytometry revealed that Flublok adjuvanted with R-DOTAP elicited functionally diverse subsets of HA-specific CD4+ T cells of greater abundance and functional diversity than that elicited by MF59 mimetic, even administered with the TLR-9 agonist CpG.

## 2. Materials and Methods

### 2.1. Mice and Ethics Statement

C57BL/6 (“B6”) female mice were purchased from the National Cancer Institute (NCI, Fredrick National Laboratory, Bethesda, MD, USA). Mice were typically used between the ages of 2.5 and 5 months and were housed in a specific pathogen-free facility at the University of Rochester Medical Center as required by institutional guidelines. The mouse experiments followed AAALAC International, the Animal Welfare Act, and the PHS Guide and were approved by the University of Rochester Committee on Animal Resources, Animal Welfare Assurance Number A3291-01. The protocols for the described studies were originally approved on 4 March 2006 (protocol no. 2006-030) and were evaluated and re-approved every 36 months. The most recent review and approval was on 21 December 2023.

### 2.2. Preparation of R-DOTAP Nanoparticles and Vaccine Formulations

Current good manufacturing practice–grade (CGMP) R-DOTAP was provided by PDS Biotechnology Corporation, Florham Park, NJ, USA. Concentrated Flublok vaccines in PBS buffer were diluted with 280 mM sucrose. Before administration to animals, the vaccines were brought to room temperature. Using a pipette to form a uniform suspension, the Flublok vaccines were added at a 1:1 ratio with the R-DOTAP nanoparticles (6 mg/mL in 280 mM sucrose), as previously described [59]. For vaccination of all cohorts of mice, a 100 μL volume was used for each dose, delivered subcutaneously in 50 μL aliquots to each of the rear footpads.

### 2.3. Influenza Infections

For H3 peptide CD4+ T cell epitope mapping, C57BL/6 mice received virus at a dose of 300 or 900 PFUs of mouse-adapted A/Switzerland/9715293/2013 (H3N2) [64], kindly provided by Florian Krammer, Mt. Sinai University, NY. Mice were anesthetized by intra-peritoneal injection with tribromoethanol (Avertin 14 μL/mg body weight) and virus, which was adjusted to 30 μL using PBS and delivered through intranasal instillation.

### 2.4. Proteins and Peptides

Fifteen-mer peptides from Influenza B Brisbane/60/08 HA were obtained from Biopeptide (San Diego, CA, USA). An Influenza A H3 A/Perth/09 peptide array containing overlapping 15-mer peptides was obtained from BEIR (BEI Resources, NIAID, Bethesda, MD, NR-19266), and single peptides (17-mers) representing divergent sequences from recently circulating H3 strains were purchased from Genscript (Piscataway, NJ, USA). A Flublok Quadrivalent (Sanofi) 2019–2020 formula containing recombinant hemagglutinin derived from the influenza strains A/Brisbane/02/2018 (H1N1), A/Kansas/14/2017 (H3N2), B/Maryland/15/2016, and B/Phuket/3073/2013 was obtained from the manufacturer through the University of Rochester Medical Center Pharmacy. Each 0.5 mL dose of Flublok contains 45 μg of each HA (180 μg total HA) in PBS and 27.5 μg of Tween-20. For the Influenza B peptide pool, three immunodominant peptides (HA-B p6/7: ^23^TSSNSPHVVKTATQGE^38^, HA-B p25: ^97^SILHEVRPVTSGCFP^111^, and HA-B p121/122: ^483^KLKKMLGPSAVEIGN^497^) were pooled (HA-B pool), with each peptide contained in the pool at a final concentration of 2 μM. For the defined H3 epitope, the two adjacent peptides, p35 and p36 (p35/36) (p35: ^203^TNNDQISLYAQASGRIT^219^, p36: ^209^SLYAQASGRITVSTKRS^225^), were pooled. In some experiments, the H3 pool was divided into peptides localized to the amino and carboxy terminal portions of the protein, and all peptides within the array and the divergent peptides were pooled and used at a final concentration of 0.5 μM. Single peptides were used at a final concentration of 2 μM.

### 2.5. Protein Immunizations

C57BL/6 (“B6”) mice were immunized with 10 μL of Flublok (3.6 μg, which is equal to 0.9 μg of each HA protein) in adjuvant using a 1:1 ratio of adjuvant and antigen diluted in an appropriate buffer. In the R-DOTAP vaccination, Flublok vaccines were diluted in 280 mM sucrose. For AddaVax (Invivogen, San Diego, CA, USA) adjuvanted vaccination, Flublok was diluted in PBS with or without CpG (2.5 μg per mouse) (ODN1826, IDT, Coralville, IA, USA), a dose that we have found to promote robust Th1 responses and that would be appropriate for a preclinical model of vaccination that would limit the pro-inflammatory responses as others have used [65,66], which is approximately 1/1000 of that used in humans. Thus, each cohort of mice received the same dose of vaccine antigen, regardless of the adjuvant system utilized. At nine days post-vaccination, the mice were euthanized, and single-cell suspensions were prepared from draining popliteal lymph nodes (pLN). CD4+ T cells were enriched by negative selection via MACS (Miltenyi Biotec, Bergisch Gladbach, Germany) using the manufacturer’s protocol. Purified CD4+ T cells were used in EliSpot assays, as described below. Alternatively, single-cell suspensions were used for ICS and flow cytometry, as described below.

### 2.6. EliSpot Assays

EliSpot assays used to quantify cytokine-secreting cells were performed as previously described [61]. Briefly, 96-well filter plates were coated with purified antibody at 2 μg/mL (IL-2: clone JES6-1A12, IFN-γ: clone AN-18, and IL-4/IL-5: clones 11B11/TRFK.5, respectively, BD Biosciences, San Diego, CA, USA) or granzyme B (1:60 dilution, 50 μL/well, R&D Systems, Minneapolis, MN, USA) in PBS overnight at 4 °C. Following incubation, the unbound antibody was removed by washing with media (complete DMEM media with 10% FBS (Fisher Scientific, Gibco, Waltham, MA, USA)). Non-specific protein binding to the plates was blocked by incubation with complete media. CD4+ T cells, isolated by MACS purification, were co-cultured with syngeneic spleen cells (500,000 per well), antigen-presenting cells (APCs), and peptide or peptide pools used at 2 μM or 0.5 μM final concentration in culture, as indicated. CD4+ T cells were plated at empirically determined concentrations to optimally enumerate mediator-producing cells (100,000–250,000 for pLN). Following 18–20 h incubation at 37 °C and 5% CO_2_, the plates were washed (1X PBS, 0.1% Tween-20) and incubated with biotinylated antibody to detect IL-2, IFN-γ, IL-4/IL-5, or granzyme B (2 μg/mL for: clone JES6-5H4, clone XMG1.2, and clones BVD6-24G2/TRFK4, respectively, BD Biosciences, and 1:60 dilution for GRZB, RD Systems) for half an hour for cytokines or 1 h for granzyme B. To the washed plates, streptavidin-conjugated alkaline phosphatase (1:1000 in wash buffer + 10% FBS, Jackson ImmunoResearch, West Grove, PA, USA) was added and incubated for 30 min at room temperature. Vector Blue substrate kit III (Vector Laboratories, Newark, CA, USA) prepared in 100 mM Tris, pH 8.2, was used for development. An Immunospot reader series 5.2 and software v5.1 (CTL, Cleveland, OH, USA) were used to quantify spots. For estimation of background responses, control cultures consisted of CD4+ T cells in media with no peptide as a negative control. All conditions were performed in duplicate or triplicate.

### 2.7. Flow Cytometry

#### 2.7.1. Peptide Stimulation for Intracellular Cytokine Staining (ICS)

To evaluate the antigen-specific response, the lymph node cells and splenocytes were washed, resuspended in RPMI primary media, and co-cultured in sterile U-bottom plates at 300,000 and 500,000 cells per well, respectively. The antigen-specific responses were determined by stimulating the cells with the HA-B pool (described above) at 1 μM per well. Plates were incubated at 37 °C and 5% CO_2_. After two hours of incubation, a combination of two protein-trafficking inhibitors, monensin (GolgiStop, BD Biosciences) and brefeldin (GolgiPlug, BD Biosciences), were added to the culture. At this time, an anti-CD107a antibody (clone 1D4B, Invitrogen, Waltham, MA, USA) was also added to the culture at a 1:200 dilution. Plates were incubated for an additional 8 h at 37 °C and 5% CO_2_ and then transferred to 4 °C overnight.

#### 2.7.2. Flow Cytometry

After stimulation (described above), the samples were transferred to V-bottom tissue culture plates and washed twice with 1xPBS. Cells were stained for viability with LIVE/DEAD Fixable Blue Dead Cell Stain (Fisher Scientific, Invitrogen, L23105) for 30 min at 4 °C. Next, the cells were washed twice with FC stain buffer (1xPBS, 2% heat-inactivated FBS, 0.01% NaN_3_) and then resuspended in anti-CD16/CD32 (FC Block, clone 2.4G2, BD Biosciences) for 15 min at 4 °C. Without washing, the cells were stained for 30 min at 4 °C with a surface stain master mix containing the following antibodies, each at a 1:200 dilution: CD4 (RM4-5, BD Biosciences), CD3 (145-2C11, BD Biosciences), CD8a (53-6.7, BioLegend, San Diego, CA, USA), B220 (RA3-6B2, BioLegend), and CD44 (IM7, TONBO, Cytek, Bethesda, MD, USA). The cells were washed twice with FC stain buffer. For the intracellular staining, cells were fixed and permeabilized using FoxP3 Fixation/Permeabilization buffer (Fisher Scientific, eBiosciences) and placed at 4 °C for 1 h. After washing the cells twice with FoxP3 wash buffer (eBiosciences), the cells were incubated in FC Block and an intracellular stain master mix containing the following antibodies at a 1:100 dilution for 1 h at 4 °C: IL-17A (TC11-18H10, BD), IL-4 (11B11, BD), IL-5 (TRFK5, BioLegend), IFN-γ (XMG1.2, BD), GrzB (GB11, BioLegend), and IL-2 (JES6-5H4, BioLegend). The cells were washed twice with FoxP3 wash buffer and then fixed in 1% paraformaldehyde for 10 min at 4 °C. Once fixed, the cells were washed and resuspended in FC stain buffer for data acquisition.

#### 2.7.3. Data Acquisition and Analysis

Samples were run on a 5-laser, Cytek Aurora (Cytek, Bethesda, MD, USA). Data were analyzed using FlowJo software, version 10.9.0 (Becton, Dickinson and Company, Ashland, OR, USA). A statistical analysis was conducted using GraphPad Prism software, version 10. Specific statistical tests used for each data set are indicated in the figure legends. *p* values are indicated as an asterisk using the following criteria: *p* > 0.05, *: *p* ≤ 0.05, **: *p* ≤ 0.01, ***: *p* ≤ 0.001, and ****: *p* ≤ 0.0001.

## 3. Results

### 3.1. Analyses of Epitope-Specific CD4+ T Cells Elicited by Flublok with Added Adjuvants Reveal Diverse Functionality of HA-Specific CD4+ T Cells

**Figure 1:**
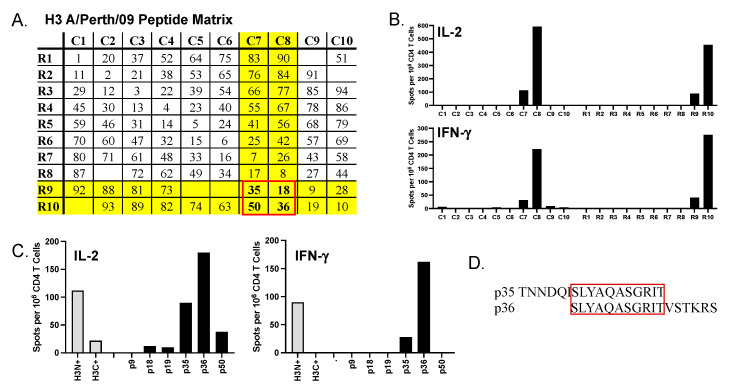
**Mapping of H3 CD4 T cell epitopes in B6 mice.** Shown in (**A**) is peptide matrix, with all peptides that collectively span H3 (*H3N2 Perth2009*) contained in discreet pools of 9-10 peptides indicated by R1 though R10 or C1 through C10. After priming B6 mice by infection with A/Switzerland 2013 H3N2 at 900 PFU/mouse, day 12 post infection CD4 T cells isolated from the spleen were tested for recognition of the peptides in each pool using a cytokine EliSpot assay (**B**) for IL-2 (top) or IFN-γ (bottom). Peptides in pools C8 and R10 were the most stimulatory followed by C7 and R9. The candidate peptides are highlighted in (**A**), with the major candidates boxed in red outline. Peptide 18 and 50 were eliminated in a subsequent assay, shown in (**C**) in IL-2 (left) and IFN-γ (right) EliSpots. Shown in (**D**), red box, is the likely core of the dominant H3 peptide, contained primarily in peptide 36.

In the experiments reported here, we studied the impact of adjuvants in the primary response to a licensed recombinant protein-based vaccine with a focus on CD4+ T cell response magnitude and functionality. The vaccine tested was quadrivalent Flublok, which offers both B cell and CD4+ T cell epitopes to H1, H3, and HAB. To quantify the impact of adjuvants on HA-specific CD4+ T cell responses to the Flublok vaccine, it was necessary to identify single-peptide epitopes that could be tracked during the response. Previous studies of ours showed that in the B6 mouse model there were no significant H1-derived CD4+ T cell epitopes detectable [67,68], as shown in Supplementary Figure S1. In our previous studies of influenza infection [67] and recombinant protein vaccination [61] in the B6 mouse strain, we defined three major HA-B epitopes (see Materials and Methods). Epitope discovery for the H3 protein in B6 mice was accomplished via infection and confirmed by infection and vaccination. Infection of B6 mice with H3N2 (A/Switzerland/9715293/2013), coupled with peptide matrix mapping [68,69,70], revealed a likely narrow CD4+ T cell peptide epitope diversity (Figure 1A,B). The H3-derived epitopes were concentrated in the amino terminus of H3 (Figure 1C, grey bars), and from this, we identified one major epitope that spanned two peptides (Figure 1D). Thus, the response of B6 mice to H3 is focused on a single major epitope, for HA-B three major CD4+ T cell epitopes, and none in H1.

**Figure 2:**
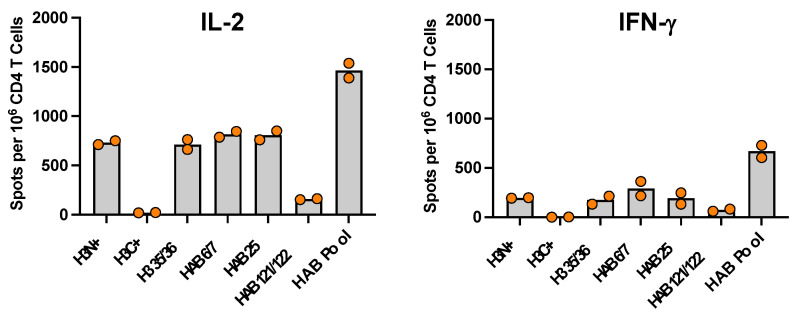
**Cytokine EliSpot assays test the specificity of CD4 T cells elicited by Flublok adjuvanted with R-DOTAP.** Mice were vaccinated with Flublok adjuvanted with R-DOTAP in the rear footpad and at day 9 the draining lymph node cells were enriched for CD4 T cells. Candidate peptides or peptide pools were tested for their ability to stimulate the CD4 T cells for production of IL-2 (**left**) or IFN-γ (**right**). The points represent the data from two independent assays and the average is shown the by the grey bar. H3N+ is a pool of peptides from the amino terminal half of the H3 Perth 2009 with added H3 strain variant peptides and the H3C+ is the comparable pool of peptides from the COOH terminus of H3, demonstrating that the defined H3 35/36 peptide comprises the primary epitope specificity. Also shown are the CD4 T cell responses to the 3 HA-B peptides previously defined.

In the first series of experiments to explore the impact of adjuvants on the response to Flublok vaccination, CD4+ T cells isolated from the draining lymph nodes of mice vaccinated with Flublok adjuvanted with R-DOTAP were tested for reactivity to and cytokine production elicited by the individual peptide epitopes from HA-B and H3. Figure 2 shows the CD4+ T cell responses in CD4+ T cells isolated from two independent cohorts of B6 mice that included two cytokines (IFN-γ and IL-2). These data show, first, that R-DOTAP reproducibly and robustly elicits CD4+ T cells specific for the known epitopes in the vaccine, including the two major epitope specificities in HA-B (HA-B p6/7 and HAB p25, with a minor contribution of HA-B p121/122) and the single H3 epitope specificity encompassed by H3 p35/36. The comparison responses stimulated by a H3 peptide pool containing all peptides in the entire amino terminal half of the H3 protein show that in CD4+ T cells, the response to H3 is completely composed of the 35/36 peptide. The greater overall CD4+ T cell responses to HA-B in response to Flublok, relative to H3, may be because the Flublok vaccine is quadrivalent, containing twice as much HA-B as H1 and H3. 

To place these responses to vaccination in the context of other adjuvants, as well as to explore the impact of them on protective effector functions, the primary CD4+ T cell response to the Flublok vaccine was examined more comprehensively, comparing the responses elicited by Flublok adjuvanted with R-DOTAP to another adjuvant system used in humans. AddaVax was included as a key comparator because it is a commercially available squalene-in-water formulation analog of MF59 [71], which has been shown to promote antibody responses in humans [72,73]. AddaVax has been used with promising results in preclinical animal models of vaccination [30,74,75,76,77]. We also analyzed the impact of AddaVax with added CpG, which is shown to promote a vaccine response that potentiates the response via engagement of TLR-9 and, thus, can promote a Th1 CD4+ T cell response [78]. This subset of CD4+ T cells is thought to provide protective immunity to many pathogens [79]. To examine the impact of these adjuvant systems on the overall CD4+ T cell response magnitude and functionality, cohorts of mice were also vaccinated with Flublok with no added adjuvant. B6 mice were vaccinated in the footpad and then examined for CD4+ T cell responses on day 9 after the primary vaccination. We also extended our analyses of CD4+ T cell responses to include IL-4 and IL-5 (combined together), Th2 cytokines that regulate B cell responses, affinity maturation, and B cell memory during cognate interactions [80], and the cytolytic mediator granzyme B, which can kill virally infected cells and mediate other anti-viral protective mechanisms [81,82].

**Figure 3:**
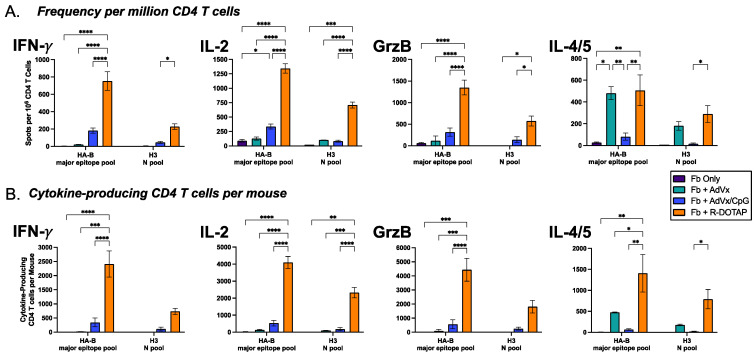
CD4 T cell magnitude and phenotype post-vaccination with Flublok with R-DOTAP or comparator adjuvant systems. Different cohorts of mice were vaccinated with Flublok adjuvanted with Addavax (turquoise), Addavax+CpG (blue), R-DOTAP (orange) or no adjuvant (purple) as indicated in the key. Elicited CD4 T cells specific for HA-B or H3 (as indicated below each panel) were quantified by EliSpots for IFN-γ, IL-2, Granzyme B, or IL-4/5, as indicated above each panel. The frequency of reactive CD4 T cells per million cells is shown in (**A**) and the sum of the frequency per mouse is indicated in (**B**), which accounts for the yield of CD4 T cells elicited by the different adjuvant systems. Shown are the average (Mean) responses with error bars indicated the standard error of the mean. Statistical values were calculated using two-way ANOVA.

Shown in Figure 3 are the results of these assays, where pools of peptides were used to maximally stimulate the elicited HA-specific cells by adjuvants. This allowed conservation of cells because of the extremely low yields of CD4+ T cells from Flublok administered without adjuvant or with AddaVax alone. The top panel (A) represents the frequency of peptide-reactive cells per million CD4+ T cells that produce IFN-γ, IL-2, IL-4/5, and granzyme B. Strikingly, R-DOTAP (shown in orange bars) elicits robust CD4+ T cells specific for H3 or HA-B for each of the mediators tested. These responses far exceeded those observed in the absence of adjuvant (in purple, Fb alone) or with Flublok/AddaVax (green bars, Fb + AdVx) or Flublok/AddaVax/CpG (blue bars), where the CD4+ T cells post-vaccination are almost undetectable. Only IL-4/5-producing cells were somewhat comparable in abundance when CD4+ T cells were elicited by R-DOTAP responses, relative to those elicited by AddaVax/Flublok. Interestingly, upon the addition of CpG to AddaVax, the IL-4/5-producing cells were diminished (see green bars), while the frequency of IFN-γ-producing cells became detectable. Both of these results are consistent with the role that CpG is known to play in promoting IL-12 and IL-18 and, thus, Th1 responses and, in parallel, diminishing Th2 responses [83,84,85,86,87,88,89,90,91,92,93]. Remarkably, beyond cytokines as a marker of CD4+ T cell function, R-DOTAP elicited a robust cytotoxic CD4+ T cell response, characterized by the secretion of granzyme B, which is the key cytotoxic mediator for CD4+ T cells. 

In Figure 3B, the total number of elicited CD4+ T cells per mouse in the vaccine-draining lymph node is shown. We calculated this because the yield of CD4+ T cells elicited by the vaccine/adjuvant or no adjuvant was different. Therefore, to represent the total HA-specific CD4+ T cells elicited that produced any given mediator, the frequency of cytokine-producing cells per million CD4+ T cells was multiplied by the total yield of CD4+ T cells per mouse that was isolated from the vaccine-draining lymph node. Thus, for example, the frequency of IFN-γ-producing cells specific for HA-B peptides that were elicited by R-DOTAP-adjuvanted Flublok (in orange) was approximately 4× that of AddaVax +CpG (in blue), and when the yield of CD4+ T cells was factored in, the relative difference was approximately 7× due to the fact that the R-DOTAP adjuvant elicited approximately twice as many CD4+ T cells compared to the AddaVax with CpG.

### 3.2. Flublok Adjuvanted with R-DOTAP Elicits Multifunctional HA-Specific CD4+ T Cells

After the discovery of the array of soluble mediators produced by CD4+ T cells elicited by Flublok adjuvanted with R-DOTAP, it was important to understand if these mediators were produced by distinct subsets of CD4+ T cells or if there was a single vaccine-induced population of CD4+ T cells. To address this, we used intracellular cytokine staining (ICS), which allows for the simultaneous detection of different mediators and cellular surface markers in single cells. Mice were vaccinated as before with Flublok with the added R-DOTAP or with the AddaVax/CpG combination, both found by the EliSpot assays above to elicit a diverse set of mediators. The draining lymph node cells were assayed by peptide-stimulated intracellular cytokine staining using the curated set of HA-B peptides shown in Figure 2 and antibodies specific for IL-2, IFN-γ, IL-4/5, and granzyme B. The combination of antibodies of both IL-4 and IL-5 was used in both the EliSpot assay and in the ICS experiments to increase the relative signal to detect Th2 cells, which can produce both mediators [94]. No co-stimulatory antibodies were included in the stimulation assay, as is sometimes used to enhance activation of CD4+ T cells [95,96]. After stimulation for 2 h and blocking of cytokine secretion for the remaining 6 h (see Materials and Methods), CD4+ T cells were permeabilized and assayed for the expression of cytokines as well as cell surface proteins (see Supplementary Figure S2 for data on the panel of antibodies and gating strategies). The responses of single animals were analyzed to apply statistical treatment to the data.

**Figure 4:**
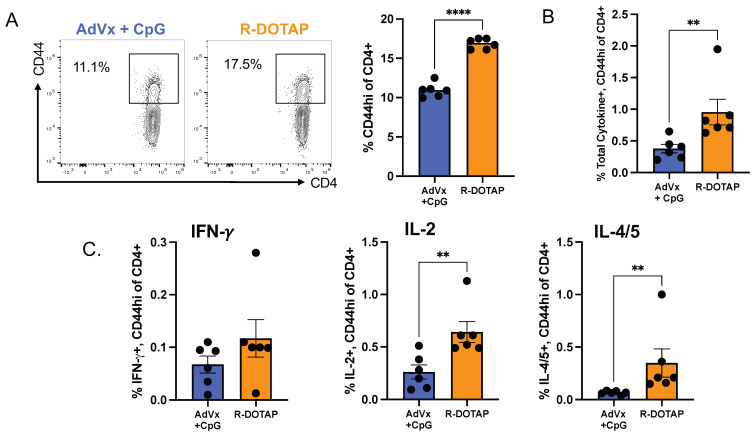
**Flow cytometry analyses to quantify vaccine adjuvant-elicited CD4 T cells.** After administration of Flublok adjuvanted with either AddaVax+CpG (AdVx+CpG), indicated in blue, or R-DOTAP, indicated in orange (6 mice per group) draining lymph node cells were sampled for expression of CD44 (as an indicator of antigen experience) or cytokine production, as described in Material and Methods, after stimulation with the pool of HA-B peptides. ((**A**), left) shows the Flow cytometry plot from a representative sample that illustrates the gating scheme used to quantify CD44 expressing CD4 T cells and the frequency of antigen experienced CD44 CD4 T cells ((**A**), right). In (**B**) the frequency of total antigen-experienced (CD44hi), cytokine producing cells were quantified by first gating on CD4, CD44 high and then the sum of those that produced either IFN-γ, IL-2 or IL-4/5. (**C**) CD44 high, CD4 T cells elicited by AddaVax+CpG (blue) or R-DOTAP (orange), were quantified for expression of the individual indicated cytokines. Shown are the individual mice (circles), mean response (bars) and error bars indicating standard error of the mean. Statistical analysis was done using the Mann Whitney unpaired nonparametric t test.

Figure 4 shows the results of the flow cytometry experiments. Panel A shows the percentage of lymph node CD4+ T cells that expressed CD44 as a marker of antigen experience. Both adjuvant systems led to the localization of many vaccine-induced, antigen-experienced cells in the primary draining lymph node. The Flublok R-DOTAP-elicited CD4+ cell population expressing CD44 was found to be approximately 1.5× greater than that of AddaVax/CpG (Figure 4A). When cytokine-producing cells were summed and quantified by the ICS assay (Figure 4B), with the gating strategies for cytokine production shown in Supplementary Figure S2, it was clear that both adjuvant systems led to readily detectable cytokine-positive cells (IL-2, IFN-γ, or IL-4/5) within the peptide-stimulated CD4+ T cells. Among the total cytokine-expressing CD44-positive cells, the number of R-DOTAP-elicited CD4+ T cells was approximately 4-fold higher than AddaVax plus CpG. The individually quantified cytokine-producing CD4+ T cells, shown in Figure 4C, show both adjuvants elicit each of the three cytokines. IL-2-producing cells were the most abundant, while the frequency of IFN-γ-producing cells was the lowest. While IL-4/5 was readily detectable in the CD4+ T cells elicited by R-DOTAP-adjuvanted Flublok, those elicited by AddaVax plus CpG/Flublok were quite modest, agreeing with the results of the cytokine EliSpot assays. Overall, the results indicate that adjuvants can promote the elicitation of CD4+ T cells with diverse effector functions. Strikingly, when compared to AddaVax, even when combined with the strong TLR-9 agonist CpG, the R-DOTAP adjuvant system elicits substantially more CD4+ T cells and, among these cells, significantly greater frequencies of cytokine-producing cells.

**Figure 5:**
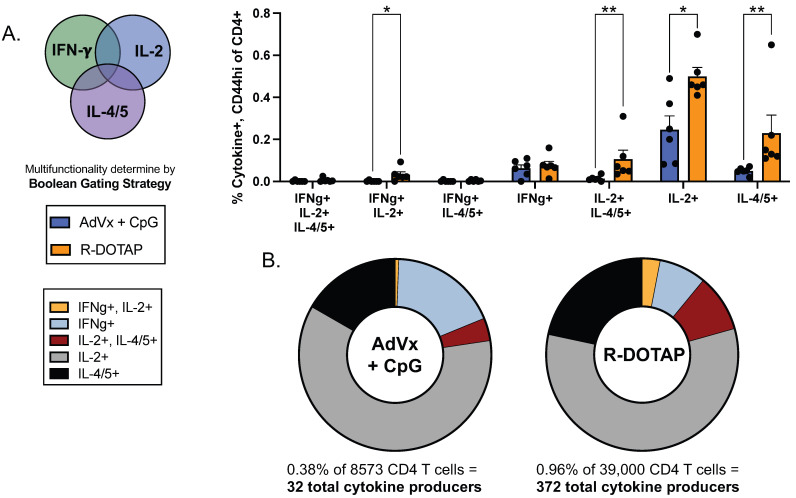
**Subsets of vaccine-elicited CD4 T cells that expressed one or more cytokines.** Two cohorts of mice (n = 6/group) were subcutaneously vaccinated with Flublok, adjuvanted with either AddaVax with CpG (AdVx+CpG; blue) or R-DOTAP (orange). Cells isolated from the draining lymph nodes were stimulated with the HA-B major epitope pool and subsequently treated to surface and intracellular staining. In (**A**), antigen experienced (CD44hi) cells expressing IFN-γ +, IL-2+ or IL-4/5+ CD4 T cells were identified and then Boolean combination gates were applied to determine the frequency of single, double or triple cytokine producers. Data is shown here as the mean ± SEM, with symbols to represent individual animals. Statistical analysis used the Mann-Whitney, multiple, unpaired, non-parametric t tests). (**B**), the frequency of total cytokine producers, listed below each pie, was normalized to 100% and each subpopulation of cytokine producers is presented as a fraction of the total. The frequency of IFN-γ +/IL-2+/IL-4/5+ triple producers and the IFN-γ +/IL-4/5+ double producers were undetectable and therefore excluded from this analysis.

We next sought to assess the subsets of vaccine-elicited CD4+ T cells that expressed one or more cytokines. Cells were gated to quantify CD4+ T cell populations producing only one cytokine (either IFN-γ, IL-2, or IL-4/5) and those producing different combinations of these cytokines. The results of these experiments are shown in Figure 5. As indicated by the frequencies shown in Figure 5A, dominant populations of CD4+ T cells elicited by Flublok and R-DOTAP produced single cytokines, either IL-2 alone or IL-4/5 alone, and among these populations, R-DOTAP produced 2 to 5 times the frequency of epitope-specific cytokine-producing cells, respectively. Also, and importantly, a readily detectable population of CD4+ T cells elicited by R-DOTAP produced both IL-2 and IL-4/5, and a smaller population of cells produced both IL-2 and IFN-γ. The number of cells producing only IFN-γ was small and similar with both adjuvant systems. In Figure 5B, these data are represented as a pie graph, which indicates the fractional distribution of the distinct cytokine-producing populations elicited by the two adjuvant systems when all the cytokine-producing cells were summed (see frequencies below each graph). When represented this way, the AddaVax with added CpG was found to elicit proportionally more CD4+ T cells that produced only IFN-γ. R-DOTAP/Flublok elicited CD4+ T cells of more diverse functionality that included a higher fraction of epitope-specific cells that produced multiple cytokines. 

### 3.3. CD4+ T Cells Elicited by Flublok-R-DOTAP Have Cytotoxic Potential and Co-Produce Effector Cytokines

**Figure 6:**
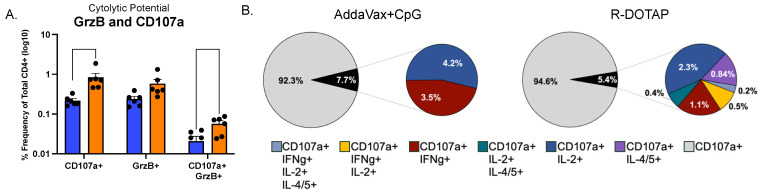
**Vaccine induced CD4 T cells with cytotoxic potential and polyfunctionality.** CD4 T cells elicited by Flublok adjuvanted with either AddaVax+CpG (blue) or R-DOTAP (orange) were stimulated with the HA-B peptide pool. They were sequentially gated on CD4+, CD44+ and then analyzed for cytotoxic activity, based expression of the degranulation marker CD107A. In (**A**), CD107a+ cells were gated for expression of Granzyme B (GrzB) or for cells expressing both, as indicated beneath each bar. (**B**). CD4+CD44+ cells CD107a+ cells were examined for the expression of IL-2, IFN-γ, IL-4/5 or combinations of these cytokines, using Boolean gating. The fraction of CD107a positive cells that were also making cytokines is shown first as a black slice of the pie in grey. The cytokine profiles of these cells were then quantified for the cytokines indicated in the excised pie diagram in color. The colors of these slices represent the cytokines produced by the CD107a positive cells. The statistical tests in A represents six replicate animals from each group using multiple unpaired t tests with Welch’s correction.

Finally, the phenotype of CD4+ T cells with cytotoxic potential was examined using a marker of degranulation (CD107a) [97,98] and the specific cytotoxic mediator granzyme B. Cytotoxic cells are increasingly recognized as an important mediator of protective immunity [50,99]. Figure 6 shows the quantification of the population of epitope-specific CD4+ T cells that were granzyme-positive and expressed CD107a. Cytotoxic CD4+ T cells have been shown to mediate killing by producing different granule components [49,99], and the expression of CD107a is an inclusive marker of granule exocytosis. Both adjuvants induced CD4+ T cells with these markers of cytotoxic potential, with R-DOTAP eliciting as much as four times as many as did AddaVax with added CpG (Figure 6A). When the CD4+ T cells that were CD107a+ were evaluated for production of cytokines (Figure 6B), we found that a substantial fraction of cells that expressed CD107a also produced cytokines such as IL-2, IL-4/5, and IFN-γ, either alone or in different combinations. The complexity of cytokines detected in cytotoxic cells elicited by R-DOTAP was distinct from AddaVax with added CpG. Not only were there fewer cytotoxic cells, but among those, the CD107a-positive CD4+ T cells expressed only the single cytokines IFN-γ and IL-2. However, the CD107a-positive cells from Flublok adjuvanted with R-DOTAP produced an array of cytokines, including some cells that produced both IL-2 and IL-L4/5 (in turquoise and IL-2 and IFN-γ in gold). Collectively, these data show that adjuvants can profoundly enhance the elicitation of HA-specific CD4+ T cells that have diverse functionality. The R-DOTAP adjuvant system was found to outperform each of the other adjuvants tested, both in terms of the total number of CD4+ T cells elicited and in terms of the complexity of functional subsets of epitope-specific CD4+ T cells primed by a single vaccination.

## 4. Discussion

The important role of CD4+ T cells as critical effectors for many pathogens has been increasingly recognized. Not only do these cells play a pivotal role in the expansion of B cells and the affinity maturation of neutralizing antibodies, but CD4+ T cells also provide direct effector function. Their secreted mediators are diverse and can foster immunity in distinctive ways that enhance the functionality of other cell types. They can also directly provide protection through the production of anti-viral cytokines and the expression of cytotoxic activity. Moreover, CD4+ T cells can provide persistent immunity in the face of antigenic drift by virtue of their recognition of small proteolytic fragments from proteins dispersed across the pathogen proteome generated within infected antigen-presenting cells or shed from infected and dying cells [40,100,101,102,103]. The importance of cross-reactive CD4+ T cells in humans has been found in many studies due to their relative broad epitope specificity. These have been noted when the novel pandemic H1N1 influenza strain emerged in 2009 [104,105] and in the recent emergence of the SARS-CoV-2 [106,107], even for receptor-binding proteins where much of the CD4+ T cell reactivity was maintained for the SARS-CoV-2 spike [108,109] as well as the more genetically conserved internal virion proteins that are not subject to antibody-mediated drift (reviewed in [40,41,107,110,111,112,113,114,115]).

Because of these contributions to persistent protective immunity to viruses and the necessity of enhancing broader and more robust antibody responses to vaccination, the studies reported here focused on the potential of the novel adjuvant R-DOTAP, when combined with a licensed influenza recombinant protein vaccine, to elicit multifunctional CD4+ T cells specific for peptides contained in recombinant HA-based protein vaccines. We compared R-DOTAP-elicited CD4+ T cells to responses elicited by other adjuvant systems, including the homolog of MF59 (AddaVax) with or without the potent TLR-9 agonist CpG. This TLR has been shown to be an effective means to promote effector CD4+ T cells. We assessed these adjuvants via a single vaccination in naïve mice in the primary vaccine-draining lymph node to gain a more comprehensive understanding of the functional potential of the elicited CD4+ T cells, which is likely to vary in different humans. 

Our studies revealed a high degree of complexity in the mediators produced by CD4+ T cells in the primary response to Flublok adjuvanted by R-DOTAP. These functions can potentiate both the primary response to vaccination and the establishment of more protective immunity to future infections. IL-2, the major cytokine elicited by R-DOTAP, has a multiplicity of functions. Although early studies pointed to its primary role as fostering the expansion of other T cells, in the decades since its discovery, it has become clear that the functions of IL-2 are much more diverse (reviewed in [116,117,118,119,120,121]). It has been shown to be critical for the establishment of CD8 T cell memory and effector function, promote the cytotoxic function of CD4+ T cells, be a key regulator of Treg function, and regulate the germinal center response through its influence on follicular helper cell differentiation. A second mediator (IFN-γ), induced by both adjuvant systems, also has a multiplicity of functions. Early studies showed the ability of this mediator to upregulate MHC class I and class II expression and activity in antigen-presenting cells [122,123] and its direct anti-viral activity, often in conjunction with innate cells and other IFN proteins, diminishing the replication of viruses [124]. Recent studies have shown that IFN-γ can promote the effector function of CD8 T cells by promoting lung-resident CD103+ CD8 T cells [125] and driving memory B cell responses in the lung [126]. In contrast to the role of IFN-γ in protective immunity through other cellular effector functions, Th2 cytokines much more robustly induced by R-DOTAP, relative to the other adjuvant systems tested, have their best recognized role in enhancing antibody response through their function in B cell expansion, germinal center responses, and B cell memory [80,127]. Thus, CD4+ T cell-secreted IL-4 and IL-5 can enhance both the primary B cell responses to vaccination and future responses to infection or vaccination. Finally, the cytotoxic potential of Flublok-elicited CD4+ T cells has been revealed by these studies. Cytotoxic CD4+ T cells have been increasingly validated as an important component of CD4+ T cell effector function [48,50,99]. CD4+ T cells expressing granzyme B, a prominent mediator in cytotoxic CD4+ T cells, were robustly elicited in HA-specific CD4+ T cells in response to Flublok/R-DOTAP and to a much greater extent than other adjuvants. Based on evidence of degranulation by expression of CD107a, additional cytotoxic mediators may also be induced in the CD4+ T cells elicited by R-DOTAP. Interestingly, a subset of CD107a-positive cells elicited by R-DOTAP also produced cytokines, including both single cytokines and combinations of cytokines. 

The events in the vaccine-draining lymph node that initiate these distinct and complementary fates of the elicited CD4+ T cells are not known at the present time. Previous studies demonstrated the induction of Type I IFN responses, including IFN-α and IFN-β, and other innate mediators in CD11c+ dendritic cells in the vaccine-draining lymph node [59]. These may play a role alone or in combination with the early T cell-derived cytokines in the differentiation of the CD4+ T cells. IL-2 and IL-15 have also been implicated in cytotoxic CD4+ T cell development [99]. Also, it has been shown in several systems that high epitope density and secondary contacts of T cells with antigen-presenting cells promote the development of cytotoxic CD4+ T cells [99,128]. These findings raise the possibility that protein uptake and persistence of peptide-bearing antigen-presenting cells may be additional components of the responses to this adjuvant system that foster greater and more complex responses. In future experiments, we plan to explore these early events in CD4+ T cell priming and vaccination that play a deterministic role in CD4+ T cell fate decisions. 

## 5. Conclusions

Many licensed and candidate vaccines against respiratory pathogens, such as influenza and SARS-CoV-2, are primarily designed and evaluated based on their ability to elicit neutralizing antibodies. However, it has now become clear that this strategy is insufficient to provide long-lived protective immunity due to the combined effects of immunodominance in the B cell responses, immune imprinting, and the rapid selective mutations and “drift” that enable the outgrowth of viral variants that have successfully eliminated recognition by these vaccine-induced neutralizing antibodies [11,129,130]. In contrast to B cells and their elicited neutralizing antibodies, CD4+ T cells provide protective immunity that has notable advantages for long-lived protection against infection. First, as shown here and in our previous studies, the epitopes recognized by CD4+ T cells are diverse, extending well beyond viral receptor sites [40,100]. Secondly, and importantly, the effector functions and homing potential of CD4 T cells are largely independent of the antigen or peptide epitopes they target [131,132]. Finally, they convey numerous effector functions that collectively promote effector functions and memory by other cells in the immune system, including B cells and CD8 T cells, as well as their own direct effector functions such as secretion of anti-viral cytokines and cytotoxicity [34,133,134]. 

In this study, we now provide strong evidence that diverse epitope distribution and effector functions by CD4 T cells can be elicited by a licensed protein-based influenza vaccine when combined with the novel adjuvant R-DOTAP. Our studies revealed that R-DOTAP promotes the elicitation of multifunctional HA-specific CD4 T cells that possess distinct and diverse effector functions, including cytotoxic activity and production of the cytokines IL-2, IL-4, and IFN-γ. The safety of R-DOTAP in humans, combined with its efficacy, offers promising options for exploring this adjuvant system for the provision of long-lived, broadly protective immunity.

## Data Availability

The full complement of data accumulated for these studies is available upon request.

## Supplementary Materials

The following supporting information can be downloaded at https://www.mdpi.com/article/10.3390/vaccines12030281/s1, Figure S1: CD4 T cell repertoire post-vaccination with Flublok with R-DOTAP or AddaVax/CpG. Figure S2: Flow cytometry panel and representative gating scheme.
