# Supplementary material for: Flublok Quadrivalent Vaccine Adjuvanted with R-DOTAP Elicits a Robust and Multifunctional CD4 T Cell Response That Is of Greater Magnitude and Functional Diversity Than Conventional Adjuvant Systems
Source: Vaccines (Basel). 2024 Mar 7;12(3):281. doi: 10.3390/vaccines12030281 (PMC10975948; doi:10.3390/vaccines12030281)
Supplement: Supplementary file 1 [file vaccines-12-00281-s001.zip › vaccines-2876902-supplementary.pdf]

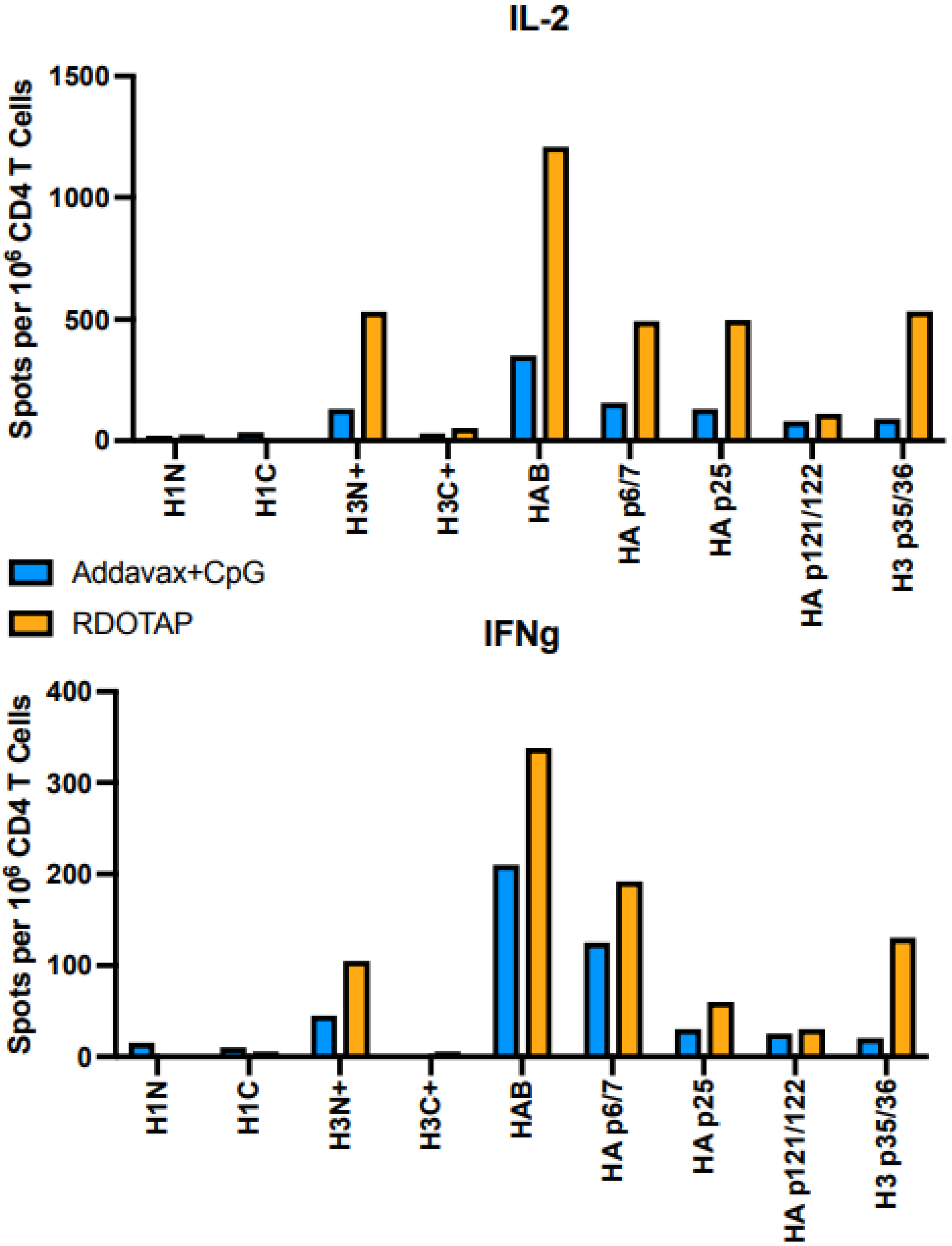

Figure S1. CD4 T cell repertoire post vaccination with Flublok with R -DOTAP or AddaVax/CpG. Different cohorts of mice were vaccinated with Flublok adjuvanted with Addavax + CpG (blue) or R-DOTAP (orange) Elicited CD4 T cells specific for pools of peptides for H1, H3 and HA -B or individual peptide epitopes identified H3 (as indicated below each panel) were quantified by IL -2 (top panel) and IFN - g (bottom panel) EliSpot assays. Shown is the frequency of cytokine producing per million CD4 T cells from the draining popliteal lymph nodes nine days post vaccination from pooled mice from a representative experiment.

A.

Antibody	Fluorophore	Clone	Company	Catalogue #
IL-17a	BUV395	TC11-18H10	BD	565246
CD4	BUV805	RM4-5	BD	741912
IL-4	BV421	11B11	BD	562915
IL-5	BV421	TRFK5	BioLegend	504311
CD3 (CD3e)	BV480	145-2C11	BD	746368
IFNg	BV786	XMG1.2	BD	563773
GrzB	FITC	GB11	BioLegend	515403
CD8a	PerCP-Cy5.5	53-6.7	BioLegend	100734
CD107a	PerCP-eFluor710	1D4B	Invitrogen	46-1071-82
B220	PE-Cy5	RA3-6B2	BioLegend	103210
IL-2	PE-Cy7	JES6-5H4	BioLegend	503831
CD44	APC-Cy7	IM7	TONBO	25-0441-U100

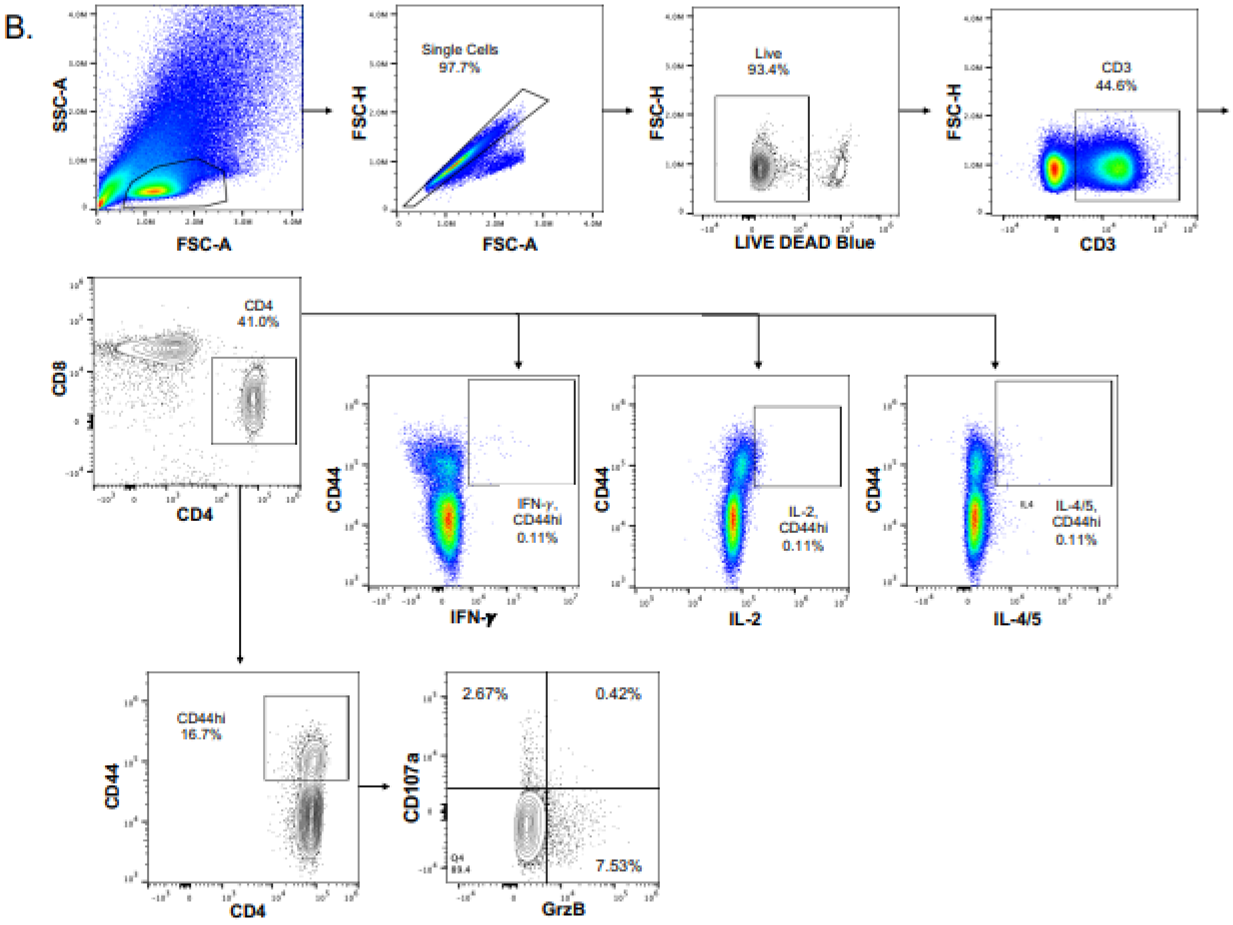

Figure S2. Flow Cytometry panel and representative gating scheme (A) All fluorescentlyconjugated antibodies utilized in the flow cytometry experiments, with catalogue numbers, have been tabulated here. (B) The following gating strategy was utilized to identify populations of antigen-reactive CD4 T cells. The analysis began by first gating on the lymphocyte population, followed by the exclusion of doublet cells. Live cells were then evaluated for their expression of CD3 and subsequently CD4. Antigen experienced, cytokine producers were identified by the co-expression of CD44 and either IFN-g, IL-2 or IL-4/5. Of note, IL-4 and IL-5-targeting antibodies were conjugated to the same fluorophore, BV421. This allowed us to capture any cells that expressed either cytokine, or both cytokines, in a single gate. CD4 T cells with cytolytic potential were determined by first gating on the antigen-experienced population, defined as CD4+, CD44hi. These cells were then partitioned via quadrant gating to determine their expression of GrzB, CD107a or both.
